# The Anatomy and Diseases of the Breast

**Published:** 1845-11

**Authors:** 


### REVIEWS.

---

Art. VIII.—*The Anatomy and Diseases of the Breast.* With numerous plates. By Sir Astley Cooper, Bart., F.R.S., Sergeant Surgeon to his Majesty; Consulting Surgeon to Guy's Hospital; Lecturer on Anatomy and Surgery, &c., &c., &c. To which are added, his *various Surgical Papers*, now first published in a collected form. Philadelphia: Lea & Blanchard. 1845.

The superb style in which this volume is brought out by the publishers is worthy of the works of the great English Surgeon, of which this forms the last of the series. As a whole, it is admirable—paper, type, and lithography. Of the matter, there is no need that we should speak. It is a part of the fruits of fifty years' observation and practice, and has all the maturity which age and experience could give it. This volume, henceforward, will take its place among the classics of Surgery.

No analysis of this work will be attempted, for we have not the space at our command, and if we had we are not sure that an abstract would be desirable. It is a production to be studied leisurely and carefully, and can only be appreciated when seen in detail. Still, limited as we are for room, we will not take leave of the volume without an extract. It relates to a practical subject, and while it conveys substantial information it affords also a sample of the direct, unaffected manner in which the author treats his subject.

"*On the Effects of Common Inflammation in the Breast.*—Having divided the Diseases of the Breast into those which are the result of common inflammation, whether acute or chronic, into those which are of a peculiar or specific nature, and into such as are specific and malignant, I shall now proceed shortly to describe the effects which are produced upon it by acute and chronic inflammation.

"It is unnecessary that I should enter into any long detail of the symptoms, character, and treatment of acute inflammation in this organ, as it differs little from the same inflammation in other parts of the body, excepting in the severity of suffering which it produces.

"It is adhesive in the first stage, suppurative in the second, and ulcerative in the third. A firm and sensitive swelling of the whole or part of the mammary gland is produced in the first stage; and the dense cellular or fascial membrane with which it is enveloped, and by which all its parts are united, not easily yielding to the inflammatory swelling, often occasions most excessive suffering. The serous and fibrous portions of the blood are poured into, and fill the interstices of the inflamed structure, and the latter thus produces the solid swelling. To this enlargement succeeds a blush of inflammation upon the surface of the breast; throbbing, pulsatory, and very acute pain follows it; a particular prominence and smoothness are observed at one part of the tumor, with a sense of fluctuation from the presence of matter. The constitution is also highly irritated, which is evinced by the occurrence of shivering, succeeded by heat and profuse perspiration. Over the most prominent part of the swelling the cuticle separates, ulceration follows in the cutis, and the matter becomes discharged through the aperture thus produced.

"This process requires from ten days to three weeks for its completion; but varies considerably in time in different persons, according to the irritability of their constitution, and to the depth at which the abscess is formed.

"Its principal cause is the rush of blood which takes place each time the child is applied to the bosom, and which by nurses is called the draught, and is the preparatory step to the secretion of milk. Such occasional irregular, violent, and frequent determinations of blood produce inflammation; and the necessary frequent exposure of the bosom in suckling, add to the occasional irregularities of the circulation. The nurse also often produces these abscesses immediately after the lying-in, by refusing to put the child early to the breast, and by stimulating the mother with strong drinks. By the first, the child is refused a secretion which precedes that of pure milk, and which often acts as a wholesome aperient to it, and the breast of the mother is excited by being permitted to remain full; whilst by the latter, the mother is heated, and a disposition to inflammation is engendered.*

"The best mode of treatment in these cases is to use, in the adhesive stage, a lotion of one ounce of spirit of wine, and five ounces of water, or of liquid plumbi dilutus to the part, and to purge the patient, by giving repeated doses of castor oil, or sulphate of magnesia. But if the patient suffer from cold produced by the evaporation of the spirit, a simple tepid poultice may be substituted for it, occasionally applying leeches to the swelling, still recollecting that the chief dependence is upon purging.

"By these means the suppurative stage will often be prevented; but if matter should form, fomentations of poppy decoction, and poultices made with the same decoction, mixed with bread, should be applied to the breast three or four times a day; as these by their warmth encourage secretion, by their moisture sooth and relax the part, and by their narcotic qualities diminish the sensibility of the nerves.

"In order still further to mitigate the sufferings of the patient, to diminish irritability and to calm the symptoms of irritation, it is necessary to give opium combined with the liquor ammoniæ acetatis, or simple saline draughts, with small doses of magnesiæ sulphas.

"It is a question with Surgeons, whether these abscesses ought to be opened by art, or be left to the processes of nature; and the following should be the reply to this query:—If the abscess be quick in its progress, if it be placed in the anterior surface of the breast, and if the sufferings which it occasions are not excessively severe, it is best to leave them to their natural course, rather than to employ the lancet for the discharge of the matter. But if on the contrary, the abscess in its commencement be very deeply placed—if its progress be tedious—if the local sufferings be excessively severe—if there be a high degree of irritative fever, and the patient suffer from profuse perspiration, and want of rest, much time is saved, and a great diminution of suffering produced, by discharging the matter by the lancet.

"Still it is wrong to penetrate with the lancet through a thick covering of the abscess, as the opening does not suc-

---

* I have heard Dr. Key say that puerperal fever is often excited by the early and injudicious use of stimulants.

ceed in establishing a free discharge of matter; for the aperture closes by adhesion, the accumulation of matter proceeds, and the ulceration will still continue: on this account, the opening should be made where the matter is most superficial, and the fluctuation is distinct, and it should be in size proportioned to its depth.

"The varieties which I have seen in these abscesses, are that several sometimes form in the same breast, quickly succeeding each other, and leading to a very protracted suffering. In these cases opium and quinine will be required to lessen irritability and support the strength of the system. Sometimes an abscess is produced at a great depth in the breast, and discharges itself by several different apertures, forming sinuses of various extent. The best mode of treatment of these cases, as far as I have had an opportunity of observing, is to inject them with a solution of two or three drops of the strong sulphuric acid to an ounce of rose-water, and to apply the same solution by folds of linen over the bosom, by which the secretion of matter is checked, and adhesion is produced.

"Now and then a deep-seated abscess forms between the posterior surface of the breast and the ribs, which, when it breaks, leaves a sinus which leads to the ribs. An exfoliation of part of the rib afterwards occurs, occasioning a very protracted suffering; and in these cases, as well as in the former, injecting the diluted acids is the best practice.

"The division of the sinus by the knife is unnecessary, as it will heal by adhesion in the former case, and in the latter, unless the exfoliating bone be loose, no advantage will be derived from the incision.

"I once saw a lady of a very delicate constitution, and who suffered under very great anxiety of mind, from her husband having been confined in prison for debt, who after her lying-in had a milk abscess in her bosom, which broke, and discharged large quantities of matter; and then, instead of healing, the whole breast became excessively swollen, and a truly fungoid excrescence appeared, by which disease she was soon destroyed.

"A hardness sometimes remains in the breast after these abscesses, which continues for a great length of time if something be not done to promote absorption; and as a morbid action will sometimes, and at a very distant period, arise in the swelling, it is a great object to dissipate it quickly; which will be best effected by the application of the Emplastrum

Ammoniaci cum Hydrargyro, or by rubbing the part with the Iodine Ointment.

"In these milk abscesses it becomes a question whether the child should suck the breast or not. If the abscess be small the child may be put to the diseased breast as well as the other; but if much of the mamma be involved in the disease, the child should be put to the other breast; but that which is inflamed should be drawn by the mother herself, by means of the glass tube constructed for the purpose. As the pump which is sometimes employed, bruises the breast, and gives much pain, it ought not to be used. As a general rule, it is best to continue the child at the breast so long as the mother's sufferings will admit of it.

"These abscesses are sometimes the result of soreness in the nipples, which appear in three forms:—first, in simple excoriation; secondly, in deep cracks at the junction of the nipple with the areola; and thirdly, in deeper ulceration of the nipple itself, by which a part of it is removed. The suffering from these sores is often sufficiently great to prevent the frequent application of the child to that bosom, which leads to a great accumulation of milk, and to a degree of distention which occasions inflammation. To prevent this, the breast should be drawn; but the sooner the child can be restored to it, the better. The best application to the sore nipple is a solution of borax in water, in the proportion of a drachm of borax, three ounces and a half of water, and half an ounce of spirit of wine. Some use solutions of alum, some the sulphate of zinc, and some the supernatant liquor of a mixture of the liquor calcis with the submuriate of mercury. Also to prevent the nipples from becoming sore, to which many women are extremely subject, it is right to wash them sometime before the lying-in with strong brine, which hardens the cuticle, and renders it less prone to ulceration and inflammation.

"When the nipple is deeply ulcerated, if the child continue to suck, it must be through the intervention of a shield prepared with the cow's teat.

"When women marry young, the nipple is often very small; the child is unable to suck it, and the attempt to do so produces excessive pain. The nurse should in this case frequently suck the breast, which she is able to do with much less pain than the child, because her mouth not only envelopes the nipple, but a large portion of the breast around it.

"Ointments in general do not agree as applications to these sores; but if any be employed under a shield, it should be the ointment of bismuth, or the ointment of zinc, or simple cerate.

"*Of Chronic Abscesses.*—The abscesses which I have hitherto described, usually pass through their different stages in from three to five weeks; but under the chronic inflammation, an abscess is sometimes produced, which, from the length of time it is forming, from the little pain which attends it, from the absence of redness, and heat in the part, and from the want of rigors and other constitutional symptoms, prevent the suspicion of the formation of matter, and the swelling is supposed to be a malignant tumor which requires an operation for its removal.

"In proof of this, a woman was sent to me from Sussex who had a tumor in her breast, which I was requested to remove; and when she was seated before me for that purpose, I found upon examining the swelling with attention, a fluctuation in its centre surrounded by a wall of hardness, with tenderness in the centre upon pressure. I therefore put a lancet into the seat of the fluctuation, to discover the nature of the fluid, and a considerable quantity of purulent matter was discharged through the orifice.

"I was also requested to see an out-patient at Guy's Hospital, who had a swelling in her breast with a fluctuation in its centre, which had existed several months, into which when a lancet was put, a large quantity of matter was discharged. Although there was no discoloration, and the swelling had existed several months, yet I thought it contained matter, from the sense of fluctuation, and from the tenderness the patient expressed upon slight pressure, which would not have been the result if a serous fluid had been collected.

"In similar cases I have seen the operation for removing the swelling begun, and in its progress the knife having accidentally entered the abscess, the Surgeon by escape of the matter having been informed of his error, the operation was suspended, and a poultice being applied the case ended favorably.

"As there is some defect in the constitution, or deficiency in some of the secretions in these chronic swellings, the patient requires the pilula hydrargyri submuriatis composita at night, and the bark with soda twice or three times in the day; or the compound infusion of gentian with soda and rhubarb. Locally when in the adhesive stage, the emplastrum ammoniaci cum hydrargyro should be applied, or a solution of muriate of ammonia with rectified spirit of wine, both of which are used upon the principle of exciting external irritation, and of promoting absorption.

"When it is in the suppurative stage, the abscess should be opened, and the part be poulticed. The constitutional treatment is to consist of a generous diet, and of tonic medicines. When they ulcerate, and sinuses form, and are difficult to heal, as they are wont to be, stimulating injections must be employed, lotions of a similar kind be applied, and the general health be improved.

"I have seen in a chronic abscess in the breast, the glands in the axilla enlarged from simple irritation only, and which decreased after the disease of the breast was relieved.

"*Of the Lacteal or Lactiferous Swelling.*—A swelling is sometimes formed in the breast after a lying-in, which I have called the lacteal or lactiferous, because it arises from a large collection of milk in one of the lactiferous tubes.

"Its cause is a chronic inflammation in one of the lactiferous tubes near the nipple, by which its aperture becomes closed, and the tube oliterated to the extent of an inch or more.

"The patient applies to the surgeon sometime after delivery with a swelling in the breast; unpreceded by the symptoms of abscess, it distinctly fluctuates, and she complains exceedingly of a sense of distention in the part; and when the child is put to the breast to relieve it, the pain and distention are increased by the draught of milk which enters the breast so soon as the child begins to suck. The swelling is confined to one portion of the breast, from the nipple to the circumference of the organ, and it gives a distinct sense of fluctuation. The cutaneous veins are very large, but the part is otherwise undiscolored. If a lancet be passed into the swelling, several ounces of milk are discharged; and the milk being suffered to rest for a few hours, forms a cream upon its surface. If a slight puncture only be made, the milk be discharged, and the opening suffered to immediately close, the accumulation recommences, and in a short time the same appearances and sufferings are renewed.

"When the distention of the swelling is excessive, it sometimes ulcerates, and discharges the milk which it has contained, by a small aperture at a little distance from the nipple; and the opening so produced often continues through the whole period of suckling, the milk being lost, from the aperture not being received into the child's mouth; and this opening is difficult to heal, until by weaning the child, and by purges, the the secretion of milk be entirely checked.

"The treatment which this case requires is as follows:—If the mother be prevailed upon to wean her child, as the secretion of milk will soon cease in this obstructed part as in the other parts of the breast, a simple puncture will suffice to relieve the distended tube of the milk which it contains.

"But if the child still continue at the breast, the opening may be made larger, and the milk be suffered to escape at the artificial aperture, whilst the child is sucking; thus imitating the natural relief which the ulcerative process sometimes produces, until the secretion of milk ceases, from the weaning of the child, and from purges to the mother.

"In general the surgeon is consulted in this disease in a few weeks after the birth of the child; but in the following case I did not see it until twelve months after delivery.

"Mrs. Reddle, æt. 38, has a swelling in her right breast, which appeared one month after the birth of her last child; it has now existed twelve months. I opened it with a lancet and discharged six ounces of white curd mixed with a little yellow serum. The skin was undiscolored, and her general health good. She had an abscess in the breast in her former pregnancy; she had milk in her breast after delivery; and the child is thirteen months old. The present swelling began in her last confinement, and it gradually increased until it became the size of an orange, with occasional trifling pain.

"Feeling an obscure fluctuation, I discharged a saucer full of milk, like curd or clouted cream: the discharge continued for three days, and then ceased. She attributed the obstruction to a blow.

"This disease resembles in its nature the Ranula, excepting in the fluid secreted. The one is an obstruction of the submaxillary duct, and accumulation of saliva. The other is an obstructed lactiferous tube, which is followed by an immense collection of milk, from its escape being prevented at the nipple, owing to the obliteration of the duct at that part."

